# Phylogenetic Relationships and Genetic Diversity of Thai *Nepenthes* (Nepenthaceae) Revealed by Integrative Molecular Analyses

**DOI:** 10.3390/plants15142238

**Published:** 2026-07-22

**Authors:** Yaowaphan Sontikun, Sunya Nuanlaong, Tim Böhnert, Maximilian Weigend, Potjamarn Suraninpong

**Affiliations:** 1 Faculty of Innovative Agriculture, Fishery and Food, Prince of Songkla University, Surat Thani 84000, Thailand; yaowaphan.s@psu.ac.th; 2 School of Agricultural Technology and Food Industry, Walailak University, Nakhon Si Thammarat 80161, Thailand; sunyaton@gmail.com; 3 Bonn Institute for Organismic Biology (BIOB), University of Bonn, 53115 Bonn, Germany; tboehnert@uni-bonn.de (T.B.); mwei@uni-bonn.de (M.W.); 4 Herbology Research Center, Walailak University, Nakhon Si Thammarat 80161, Thailand

**Keywords:** molecular phylogeny, carnivorous plants, species delimitation

## Abstract

Accurate assessment of phylogenetic relationships is essential for understanding the evolution, taxonomy, and conservation of the carnivorous genus *Nepenthes*. In Thailand, species delimitation remains challenging because of extensive morphological variation and the occurrence of several closely related taxa. This study integrated genome-wide single nucleotide polymorphisms (SNPs) generated by Genotyping-by-Sequencing (GBS), chloroplast trnK intron sequences, nuclear ITS sequences, and AFLP markers to investigate phylogenetic relationships and genetic diversity among Thai *Nepenthes* taxa. Genome-wide SNP analysis recovered three principal clades, whereas trnK and ITS datasets provided complementary resolution at deeper and intermediate phylogenetic levels. The molecular datasets revealed broadly congruent phylogenetic patterns and improved resolution among several closely related Thai taxa. AFLP analysis of *Nepenthes mirabilis* populations revealed moderate polymorphism despite relatively high overall genetic similarity. Across all datasets, *N. mirabilis* var. *globosa* was consistently recovered within the broader *N. mirabilis* lineage and showed limited genetic differentiation from sampled *N. mirabilis* accessions. These findings provide an integrated molecular framework for understanding phylogenetic relationships among Thai *Nepenthes* and support future taxonomic, evolutionary, and conservation studies in the region.

## 1. Introduction

The genus *Nepenthes* L. (Nepenthaceae), commonly known as tropical pitcher plants, comprises a remarkable lineage of carnivorous plants characterized by highly modified pitcher-shaped leaves that capture and digest animal prey, allowing them to thrive in nutrient-poor environments. The genus is distributed predominantly throughout Southeast Asia, with additional representatives occurring in Madagascar, Sri Lanka, India, Australia, and numerous oceanic islands. Owing to its extraordinary morphological diversity, ecological specialization, and fragmented geographic distribution, *Nepenthes* has become an important model for studies of plant evolution, speciation, and biogeography [1,2]. Although nearly 300 species names have been published within the genus, current taxonomic estimates recognize approximately 160–180 extant species worldwide, with species diversity particularly concentrated in the Malay Archipelago and Indochina [1,2].

**Figure 1:**
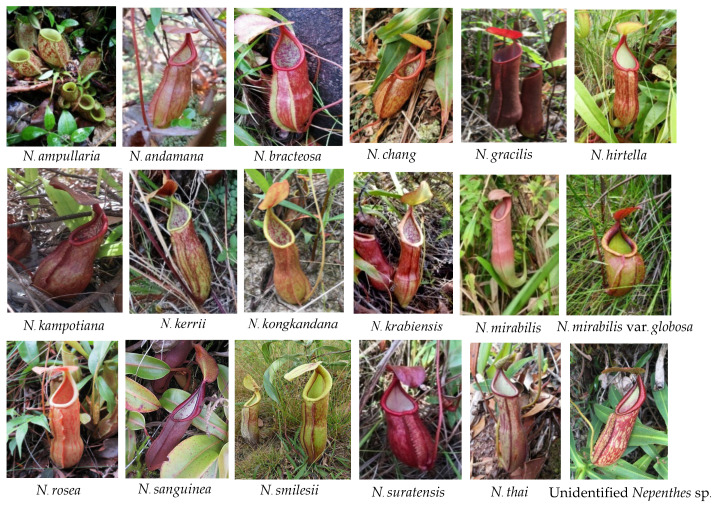
Representative *Nepenthes* taxa recorded in Thailand and included in the present study. The images illustrate the morphological diversity of Thai *Nepenthes*, including widespread species, geographically restricted taxa, and members of the *N. mirabilis* complex. Photographs were taken by Potjamarn Suraninpong and Sanya Nuanlaong unless otherwise indicated. Images of *N. smilesii* and *N. sanguinea* were adapted from photographs by François Mey (CC BY 3.0) and David Tan (CC BY-SA 3.0), respectively.

Thailand represents a biogeographically important region within the distribution range of *Nepenthe*s, serving as a transition zone between the floristic elements of mainland Indochina and the Sunda Shelf [1]. Currently, 16 species and one variety are recognized from Thailand, including both widespread taxa and geographically restricted lineages (Figure 1) [3]. Several Thai taxa, particularly members of the Indochinese *Nepenthes* complex commonly referred to as the “Tiger Group” and the *Nepenthes mirabilis* complex, exhibit substantial morphological variation and overlapping diagnostic characters, which have complicated species identification and taxonomic circumscription [4,5]. Furthermore, phenotypic plasticity, ecological adaptation, and natural hybridization contribute to taxonomic uncertainty, resulting in contrasting taxonomic treatments among authors. Such discrepancies are especially evident within the so-called “Thorelii aggregate”, where some studies have recognized multiple distinct species based on morphological characters, whereas others have adopted broader taxonomic concepts [4,5]. These persistent uncertainties highlight the need for comprehensive molecular evidence integrating genome-wide and locus-specific markers to evaluate phylogenetic relationships and taxonomic affinities among Thai *Nepenthes* taxa.

Previous molecular investigations have substantially improved our understanding of evolutionary relationships within *Nepenthes*. Early phylogenetic studies based on chloroplast markers, including the trnK intron and matK regions, provided the first molecular framework for the genus and identified major evolutionary lineages within Nepenthaceae [6]. Subsequently, analyses of nuclear ITS sequences further contributed to species-level phylogenetic inference and taxonomic assessment [7,8]. More recently, genome-skimming and phylogenomic approaches have substantially improved phylogenetic resolution across the genus, revealing complex evolutionary patterns, including reticulate evolution and relationships among major *Nepenthes* lineages [2,9]. However, molecular sampling remains uneven across geographic regions, and several Thai taxa, particularly members of the Indochinese Nepenthes complex commonly referred to as the “Tiger Group” and the *N. mirabilis* complex, remain underrepresented in comprehensive molecular datasets. In addition, no previous study has comprehensively integrated genome-wide SNPs, chloroplast sequences, nuclear ITS data, and AFLP markers to evaluate phylogenetic relationships among Thai *Nepenthes* taxa within a single analytical framework.

A variety of molecular markers have been employed to investigate plant diversity and evolution, each providing information at different phylogenetic scales. Genome-wide Single Nucleotide Polymorphisms (SNPs) generated through Genotyping-by-Sequencing (GBS) offer high-resolution insights into genetic structure, population differentiation, and evolutionary relationships [10,11]. In contrast, Amplified Fragment Length Polymorphism (AFLP) markers provide an efficient fingerprinting approach for assessing genetic variation among closely related taxa and have previously been applied to *Nepenthes* classification and diversity studies [12,13,14]. Chloroplast markers, including the trnK intron and matK gene, have proven particularly useful for resolving deeper phylogenetic relationships and major evolutionary lineages within Nepenthaceae [6,15], whereas nuclear ITS sequences often provide greater discriminatory power at lower taxonomic levels and have been widely used in species-level phylogenetic studies of *Nepenthes* [7,8,16]. Because these marker systems differ in genomic origin, inheritance patterns, and evolutionary rates, their combined application provides an opportunity to compare complementary phylogenetic signals and to evaluate taxonomic affinities among Thai *Nepenthes* taxa using multiple independent sources of molecular evidence.

To address these knowledge gaps, this study integrated genome-wide GBS-derived SNPs, AFLP markers, chloroplast trnK intron sequences, and nuclear ITS data to investigate genetic relationships among Thai *Nepenthes* taxa. By combining multiple complementary marker systems and comprehensive sampling of Thai taxa, we aimed to (i) evaluate genetic diversity and phylogenetic relationships, (ii) compare phylogenetic patterns recovered from different molecular datasets, and (iii) assess the taxonomic affinities of selected taxa within the context of existing classifications. The resulting dataset provides a regional molecular framework for future taxonomic, phylogenetic, and conservation studies of Thai *Nepenthes*.

## 2. Results

### 2.1. GBS-Based Phylogenomic Analysis of Thai Nepenthes

GBS sequencing of the 47 high-quality *Nepenthes* samples yielded 35.372 Gb of clean data, with average quality scores of Q20 = 94.13% and Q30 = 84.88%, and GC contents ranging from 36.89% to 40.13% (Table S1). De novo assembly generated 742,845 contigs (N50 = 256 bp). Mapping rates ranged from 56.25% to 94.18%, and genome coverage exceeded 20% for all samples (Tables S2 and S3). SNP discovery identified between 182,279 and 605,465 SNPs per sample, with transition/transversion ratios ranging from 1.434 to 2.502 (Table S4).

**Figure 2:**
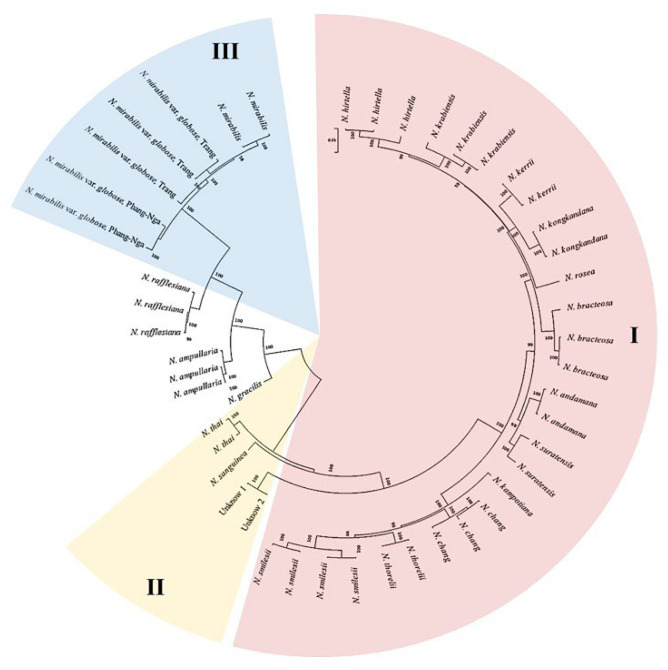
Maximum Likelihood phylogenetic tree of Thai *Nepenthes* taxa inferred from genome-wide SNPs generated by genotyping-by-sequencing (GBS). The analysis included 47 samples representing 20 taxa and recovered three major clades (I–III). Numbers at the nodes indicate bootstrap support values based on 1000 replicates.

Maximum Likelihood (ML) analysis based on GBS-derived SNPs resolved the sampled Nepenthes taxa into three major clades (Figure 2). Clade I comprised *N. bokorensis*, *N. kampotiana*, *N. chang*, *N. suratensis*, *N. andamana*, *N. kongkandana*, *N. kerrii*, *N. krabiensis*, *N. rosea*, *N. hirtella, N. bracteosa*, *N. thorelii*, and *N. smilesii*. Two major subclades were recovered within Clade I. The first subclade comprised *N. andamana*, *N. suratensis*, *N. krabiensis*, *N. kerrii*, *N. kongkandana*, *N. rosea*, *N. hirtella*, and *N. bracteosa*, whereas the second comprised *N. kampotiana*, *N. chang*, *N. thorelii*, and *N. smilesii*. Clade II consisted of *N. thai*, *N. sanguinea*, and two independently sampled individuals of an unidentified *Nepenthes* taxon (Unknown-1 and Unknown-2). Clade III comprised *N. mirabilis* and *N. mirabilis* var. *globosa*. Accessions of *N. mirabilis* from southern Thailand were recovered within the same subclade, whereas *N. mirabilis* var. *globosa* accessions from Phang Nga and Trang formed a distinct lineage within Clade III. *N. gracilis*, *N. ampullaria*, and *N. rafflesiana* were recovered as distinct lineages separate from Clades I–III.

### 2.2. Phylogenetic Analysis Based on trnK Intron Sequence

PCR amplification of the trnK intron generated fragments ranging from approximately 1100 to 1200 bp. Twenty-three newly generated trnK intron sequences were obtained from Thai *Nepenthes* taxa and analyzed together with reference sequences retrieved from GenBank. The final alignment comprised 90 taxa and 2437 nucleotide positions, with *Ancistrocladus abbreviatus* and *Triphyophyllum peltatum* designated as outgroup taxa (Figure S1).

**Figure 3:**
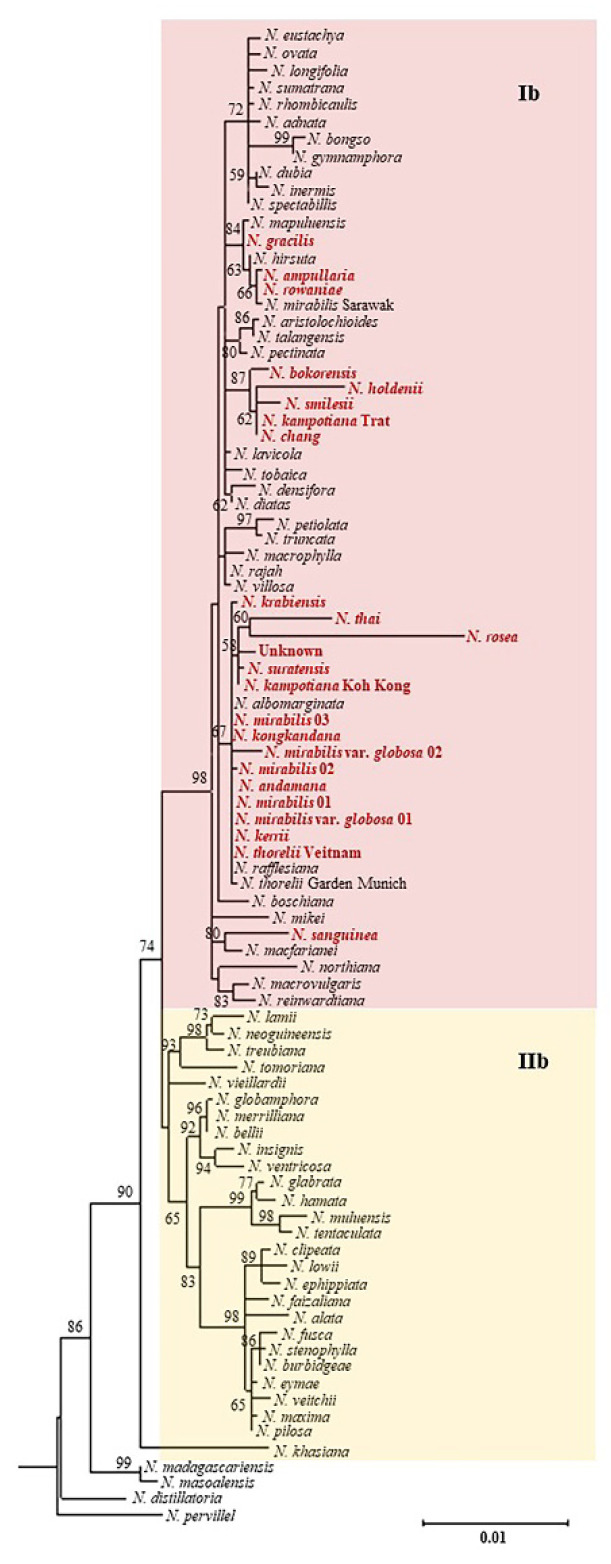
Maximum Likelihood phylogenetic tree based on trnK intron sequences of *Nepenthes*. The dataset comprised 90 taxa, including 23 newly generated sequences from Thai *Nepenthes* and reference sequences retrieved from GenBank. Four basal lineages were recovered, followed by two major lineages (Clades Ib and IIb). All sampled Thai *Nepenthes* taxa were placed within Clade Ib, whereas Clade IIb was composed predominantly of montane Bornean species. *Ancistrocladus abbreviatus* and *Triphyophyllum peltatum* were included as outgroup taxa. Numbers at the nodes indicate bootstrap support values based on 1000 replicates.

ML analysis recovered four basal lineages (Figure 3). *N. pervillei* and *N. distillatoria* occupied the earliest branching positions, followed by a strongly supported clade comprising *N. madagascariensis* and *N. masoalensis* (bootstrap = 99%), and subsequently *N. khasiana*. The remaining taxa were resolved into two principal lineages, designated Clades Ib and IIb.

Clade Ib comprised 59 taxa from Southeast Asia and Australia, including all sampled Thai taxa. Within this lineage, *N. gracilis* was recovered together with *N. hirsuta*, *N. ampullaria*, *N. rowaniae*, and *N. mirabilis* from Sarawak (bootstrap = 84%). *Nepenthes bokorensis* formed a moderately supported group with *N. holdenii*, *N. smilesii*, *N. kampotiana*, and *N. chang* (bootstrap = 87%). *Nepenthes krabiensis*, *N. thai*, *N. rosea*, and the unidentified *Nepenthes* accession were recovered within the same lineage, although relationships among these taxa were weakly resolved. All sampled accessions of *N. mirabilis* from southern Thailand were recovered within Clade Ib together with *N. mirabilis* var. *globosa* and several Indochinese taxa, including *N. andamana*, *N. kerrii*, *N. kongkandana*, and *N. thorelii*.

Clade IIb comprised 26 taxa and was dominated by montane Bornean species, including *N. veitchii*, *N. burbidgeae*, *N. stenophylla*, and *N. platychila*. Relationships among taxa within this clade were generally weakly resolved.

### 2.3. Phylogenetic Analysis Based on ITS Sequences

**Figure 4:**
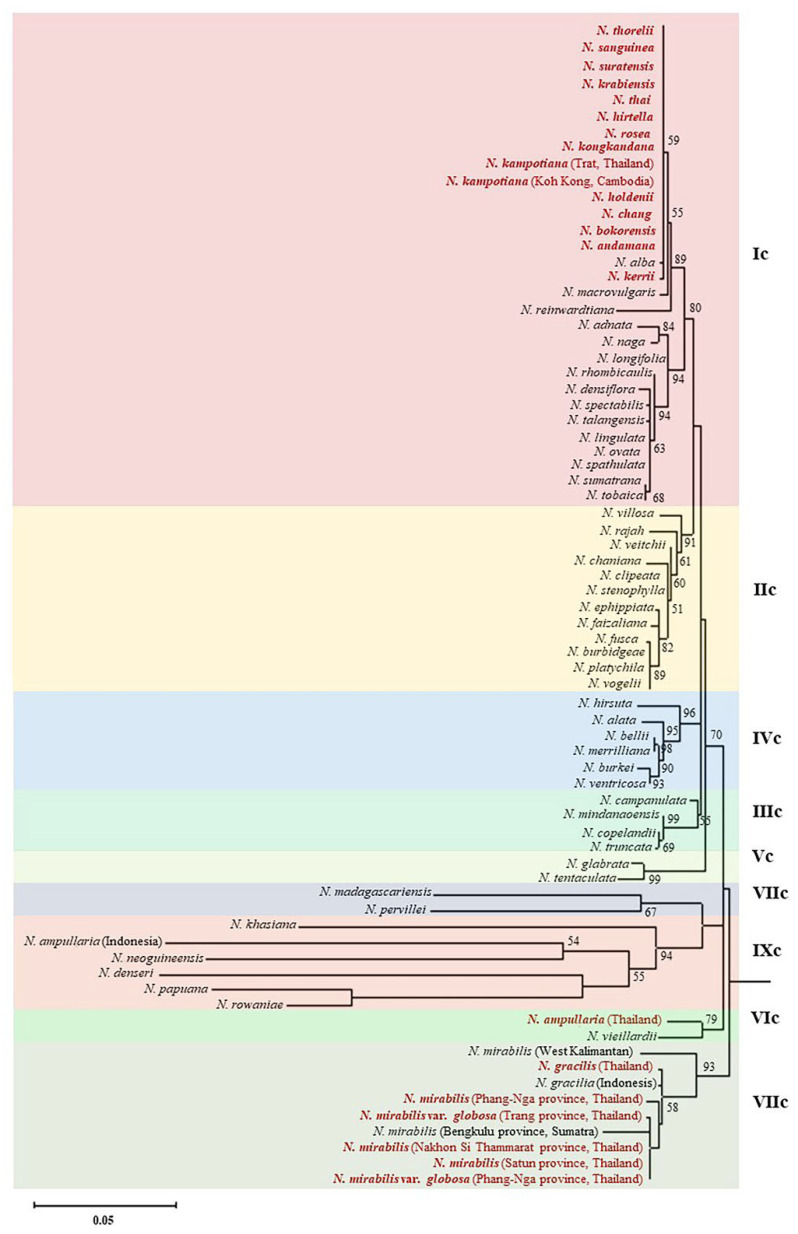
Maximum Likelihood phylogenetic tree based on nuclear ITS sequences of *Nepenthes*. The dataset comprised 75 accessions, including 24 newly generated Thai *Nepenthe*s sequences and reference sequences retrieved from GenBank. Nine major clades (Ic–IXc) were recovered. Most Indochinese taxa were grouped within Clade Ic, whereas all sampled accessions of *N. mirabilis* and *N. mirabilis* var. *globosa* were recovered within Clade VIIc. *Dionaea muscipula* was used as the outgroup taxon. Numbers at the nodes indicate bootstrap support values based on 1000 replicates.

Phylogenetic analysis of 75 *Nepenthes* accessions was conducted using an alignment of 672 ITS nucleotide positions (log likelihood = −5263.0287). ML analysis resolved nine major clades (Figure 4), with most Indochinese taxa recovered within Clade Ic.

Clade Ic received moderate bootstrap support (80%) and comprised a mixture of Indochinese taxa, including *N. thorelii*, *N. suratensis*, *N. krabiensis*, *N. hirtella*, *N. rosea*, *N. kongkandana*, *N. kampotiana*, *N. holdenii*, *N. chang*, *N. bokorensis*, *N. andamana*, and *N. kerrii*, together with *N. sanguinea* and *N. thai*. Two subclades within Clade Ic were supported by bootstrap values of 89% and 94%, respectively.

Clade IIc consisted predominantly of montane Bornean taxa, including *N. villosa*, *N. rajah*, and *N. veitchii*. Clades IIIc–Vc contained a range of Southeast Asian taxa, including *N. campanulata*, *N. mindanaoensis*, *N. hirsuta*, and *N. tentaculata*.

Clade VIc comprised *N. ampullaria* (Thailand) and *N. vieillardii*.

Clade VIIc received strong bootstrap support (93%) and contained all sampled accessions of *N. mirabilis* together with *N. mirabilis* var. *globosa*. Accessions of *N. mirabilis* from southern Thailand were recovered within the same lineage as *N. mirabilis* from West Kalimantan and Bengkulu, whereas *N. mirabilis* var. *globosa* accessions from Phang Nga and Trang were also recovered within the same lineage. *N. gracilis* was recovered as a sister lineage to Clade VIIc.

Clade IXc comprised *N. khasiana*, *N. neoguineensis*, *N. denserei*, *N. papuana*, and *N. rowaniae*.

### 2.4. Genetic Variation in Nepenthes mirabilis Revealed by AFLP and ITS Analyses

**Figure 5:**
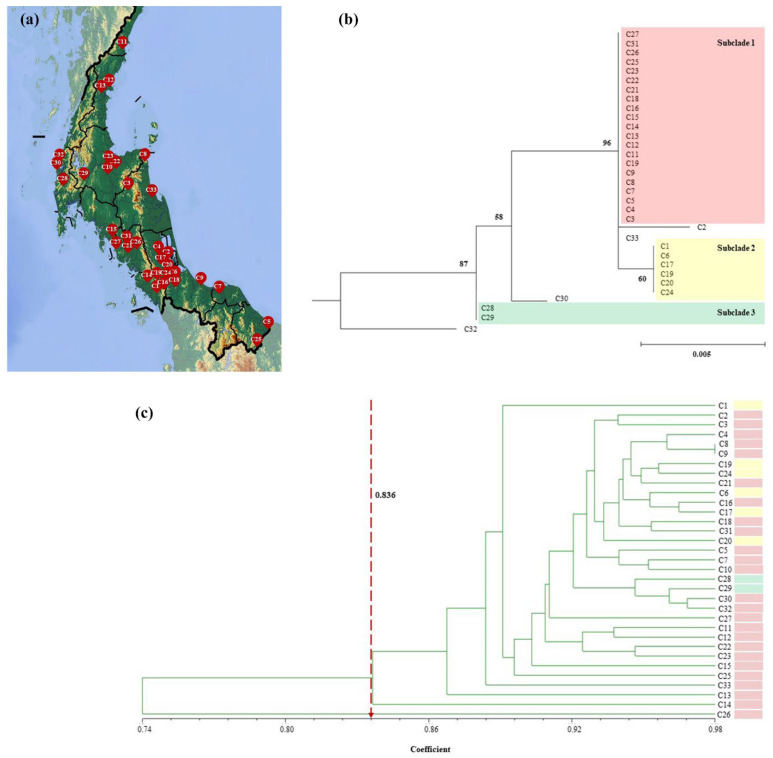
Genetic variation among 33 *Nepenthes mirabilis* accessions collected from southern Thailand. (**a**) Sampling localities of the analyzed accessions. (**b**) UPGMA dendrogram based on AFLP markers showing genetic relationships among accessions. (**c**) Maximum Likelihood phylogenetic tree inferred from ITS sequences, with *N. ampullaria* used as the outgroup. Numbers at the nodes indicate bootstrap support values.

Thirty-three *N. mirabilis* accessions collected from multiple localities across southern Thailand were analyzed using AFLP and ITS markers (Figure 5a). AFLP analysis generated 742 scorable bands using 11 selected EcoRI/MseI primer combinations, of which 323 (43.5%) were polymorphic. Polymorphic Information Content (PIC) values ranged from 0.09 to 0.27, with a mean value of 0.17 (Table 1). Genetic similarity coefficients ranged from 0.732 to 0.858, with a mean of 0.791 and a standard deviation of 0.031 (Table S5). The UPGMA dendrogram (cophenetic correlation coefficient = 0.51) indicated that most accessions formed a major cluster at a similarity coefficient of 0.836 (Figure 5b). One accession (C26) was recovered as a distinct lineage separate from the main cluster, whereas several minor subgroups and singleton accessions were recovered within the principal cluster.

ITS amplification of 33 *N. mirabilis* accessions yielded fragments of approximately 856 bp and provided sufficient variation for phylogenetic analysis. ML analysis (Figure 5c) recovered all sampled *N. mirabilis* accessions, including *N. mirabilis* var. *globosa*, within a single clade distinct from the outgroup taxon, *N. ampullaria*. No clear grouping pattern associated with sampling locality was observed within the clade.

## 3. Discussion

Integrating multiple molecular datasets, particularly genome-wide GBS-derived SNPs, provided additional phylogenetic resolution among Thai *Nepenthes* taxa and complemented evidence obtained from chloroplast and nuclear markers [8,9]. The GBS dataset recovered three principal clades, revealing patterns of genetic differentiation among major Thai lineages. Clade I was largely consistent with taxa traditionally assigned to Section Pyrophytae [17], including both peninsular Thai and Indochinese representatives. The recovery of two subgroups within this clade may reflect geographic and ecological differentiation among closely related taxa [18]. Similarly, the placement of island taxa such as *N*. *kerrii* and *N*. *chang* within broader mainland-associated lineages is consistent with previously proposed relationships among these taxa [2]. Clade II included *N*. *thai*, *N*. *sanguinea*, and an unidentified *Nepenthes* taxon, corresponding broadly to taxa traditionally associated with Section Montanae [2]. In contrast, *N*. *gracilis*, *N*. *ampullaria*, and *N*. *rafflesiana* were recovered as distinct lineages outside the three principal clades, consistent with previous molecular studies indicating substantial evolutionary divergence among major *Nepenthes* lineages [1,15].

A notable result of the GBS analysis was the close genetic association between *N*. *mirabilis* and *N*. *mirabilis* var. *globosa*. Although *N*. *mirabilis* var. *globosa* exhibits distinctive morphological characteristics [4], it was recovered within the broader *N*. *mirabilis* lineage and showed limited genetic differentiation from sampled *N*. *mirabilis* accessions. This pattern is consistent with previous AFLP and SSR studies that reported high genetic similarity among Thai *Nepenthes* populations and limited differentiation within the *N*. *mirabilis* complex [14,19]. However, additional evidence from quantitative morphological analyses, broader geographic sampling, and formal species-delimitation approaches would be valuable for further evaluating the taxonomic status of *N*. *mirabilis* var. *globosa* and related taxa within the *N*. *mirabilis* complex.

The trnK intron analysis provided a phylogenetic framework broadly consistent with previously reported relationships within *Nepenthes* [6,9,15]. Sequence lengths ranging from 2450 to 2591 bp contained sufficient phylogenetically informative variation to recover major lineages across the genus. In agreement with earlier studies, *N*. *pervillei* and *N*. *distillatoria* were recovered in the earliest branching positions, followed by the Madagascan taxa (*N*. *madagascariensis* and *N*. *masoalensis*) and *N*. *khasiana* [6,15]. The remaining taxa formed two principal lineages comprising species distributed throughout Southeast Asia, Australasia, and the Pacific region, consistent with previously published phylogenetic reconstructions [1,6,15].

Within Clade I, all sampled Thai taxa were recovered together with several Indochinese representatives, including *N*. *holdenii*, *N*. *bokorensis*, *N*. *kampotiana*, and *N*. *thorelii*. This pattern is broadly consistent with taxa traditionally assigned to Section Pyrophytae [17]. However, bootstrap support for several internal branches remained low, suggesting that relationships among some closely related taxa remain unresolved within the trnK dataset. The placement of *N*. *rowaniae* within the same broader lineage as Indochinese taxa is consistent with previously reported affinities among Southeast Asian and Australasian *Nepenthes* species [2,15].

The trnK dataset also recovered all sampled accessions of *N*. *mirabilis* within a single lineage, with *N*. *mirabilis* var. *globosa* forming a slightly differentiated branch within the broader *N*. *mirabilis* group. Similar patterns of low sequence divergence within the *N*. *mirabilis* complex have been reported previously [14,18]. Although the present results support a close evolutionary relationship between *N. mirabilis* and *N*. *mirabilis* var. *globosa*, additional evidence from quantitative morphology, population-level sampling, and formal species-delimitation analyses would be valuable for further evaluating their taxonomic relationship. Overall, the trnK results were broadly congruent with the GBS dataset, supporting several major phylogenetic patterns recovered among Thai *Nepenthes* taxa.

The ITS-based phylogeny provided additional resolution for relationships among Thai *Nepenthes* taxa and recovered nine major clades that were broadly consistent with previously published molecular studies [7,8,9,15]. Although bootstrap support was relatively low for several internal branches, a pattern commonly reported in recently diverged plant lineages [20], the ITS dataset recovered several well-supported groups and was largely congruent with the trnK and GBS analyses.

Clade I comprised primarily Indochinese taxa, including *N*. *thorelii*, *N*. *suratensis*, *N*. *krabiensis*, *N*. *hirtella*, *N*. *rosea*, *N*. *kongkandana*, *N*. *kampotiana*, *N*. *holdenii*, *N*. *chang*, *N*. *bokorensis*, *N*. *andamana*, and *N*. *kerrii*, together with *N*. *sanguinea* and *N*. *thai*. The recovery of these taxa within a common lineage is consistent with previous studies reporting close relationships among mainland Southeast Asian *Nepenthes* species [1,15]. Clades II–VIII included a range of Southeast Asian taxa representing several previously recognized lineages, particularly species distributed in Borneo, the Malay Peninsula, and other parts of Malesia [2,15]. These relationships generally corresponded with patterns reported in earlier phylogenetic studies of the genus [1,2,15].

Clade IX contained the *N*. *mirabilis* complex. Within this lineage, *N*. *mirabilis* var. *globosa* was recovered within the broader *N*. *mirabilis* lineage and exhibited limited sequence divergence from sampled *N*. *mirabilis* accessions, a pattern consistent with previous studies reporting low genetic differentiation within the *N*. *mirabilis* complex [14,18]. This result is consistent with the GBS and trnK datasets, both of which recovered a close genetic association between these taxa. Nevertheless, additional evidence from morphological, ecological, and population-level analyses would be valuable for further assessing taxonomic boundaries within the *N*. *mirabilis* complex [20,21]. Overall, the ITS results complemented the GBS and trnK datasets and supported several major phylogenetic patterns recovered among Thai *Nepenthes* taxa.

The three molecular datasets provided complementary perspectives on phylogenetic relationships among Thai *Nepenthes* taxa. Genome-wide GBS-derived SNPs provided the highest resolution among closely related taxa and revealed patterns of genetic differentiation within major Thai lineages [2,10,11]. In contrast, the chloroplast trnK intron dataset recovered deeper phylogenetic relationships and major lineages previously identified within the genus [6,9,15]. The nuclear ITS dataset provided additional resolution among closely related taxa and was broadly congruent with both the GBS and trnK analyses [7,8]. Collectively, these findings highlight the value of integrating genome-wide and targeted molecular markers to improve phylogenetic inference in taxonomically complex plant groups such as *Nepenthes* [2,9].

AFLP analysis revealed moderate levels of polymorphism despite relatively high overall genetic similarity among *N*. *mirabilis* populations [12,14]. Although most accessions formed a single major cluster, several minor subgroups and individual accessions were recovered within the dendrogram. In particular, accessions of *N*. *mirabilis* var. *globosa* from Trang and Phang Nga Provinces showed limited differentiation relative to other sampled *N*. *mirabilis* populations, consistent with previous reports of low genetic differentiation among geographically separated populations of *N*. *mirabilis* [18]. These patterns suggest the presence of genetic variation within the *N*. *mirabilis* complex while remaining consistent with the overall close genetic relationship recovered by the AFLP, ITS, trnK, and GBS datasets [14,19]. The ability of AFLP markers to detect variation among closely related populations [12,13] highlights their usefulness for examining intraspecific genetic diversity within *Nepenthes*.

In contrast, ITS sequences provided useful resolution among closely related *Nepenthes* taxa and recovered *N*. *mirabilis* as distinct from more distantly related species such as *N*. *ampullaria* [7,8,9]. However, sequence divergence among geographically proximate *N*. *mirabilis* populations was relatively limited, suggesting low levels of ITS variation within the sampled populations. Similar patterns of limited intraspecific ITS variation have been reported previously in *Nepenthes* and other recently diverged plant groups [18,20].

Both AFLP and ITS analyses consistently recovered a close genetic relationship between *N*. *mirabilis* var. *globosa* and typical *N*. *mirabilis* accessions. In both datasets, *N*. *mirabilis* var. *globosa* was recovered within the broader *N*. *mirabilis* lineage and showed limited genetic differentiation relative to other sampled populations, consistent with previous studies reporting low levels of genetic differentiation within the *N*. *mirabilis* complex [14,18]. These findings are consistent with the GBS and trnK results, which likewise recovered *N*. *mirabilis* var. *globosa* within the *N*. *mirabilis* complex. Nevertheless, because the present study did not include formal species-delimitation analyses or quantitative morphometric assessments, additional evidence would be valuable for further evaluating the taxonomic status of *N*. *mirabilis* var. *globosa* and its relationship to morphologically similar taxa, including *N*. *orbiculata* [20,22].

Taken together, the GBS, trnK, ITS, and AFLP datasets revealed largely congruent phylogenetic patterns across Thai *Nepenthes* taxa. Genome-wide SNP data provided the highest resolution among closely related taxa, whereas trnK and ITS sequences contributed information at deeper and intermediate phylogenetic levels, respectively. The concordance among independent molecular datasets provides additional support for the major phylogenetic patterns recovered in this study and highlights the value of integrating multiple marker systems when investigating taxonomically complex groups such as *Nepenthes*. The resulting molecular framework provides a foundation for future studies of taxonomy, evolution, and conservation of Thai *Nepenthes*.

## 4. Materials and Methods

### 4.1. Plant Materials, DNA Extraction, and GBS Analysis

A total of 98 individuals representing 20 taxa were initially collected. Following DNA quality and quantity assessment, 47 samples meeting the required criteria were selected for GBS library preparation and sequencing. The dataset comprised 16 recognized Thai *Nepenthes* species, one variety (*N*. *mirabilis* var. *globosa*), one unidentified *Nepenthes* taxon represented by two biological replicates, and two comparative taxa (*N*. *rafflesiana* and *N*. *thorelii*). Each taxon was represented by three to five independently sampled individuals. Two geographically distinct populations of *N*. *mirabilis* from Satun and Nakhon Si Thammarat provinces were included to assess intraspecific variation. Detailed information on sampled taxa, collection localities, and sample codes is provided in Table S6.

Genomic DNA was extracted from approximately 1 g of young leaf tissue using a modified CTAB method [23]. DNA quality was assessed by agarose gel electrophoresis and NanoDrop 2000 spectrophotometer (Thermo Fisher Scientific, Waltham, MA, USA), while DNA concentration was quantified using a Qubit 2.0 fluorometer (Thermo Fisher Scientific, Waltham, MA, USA) prior to library preparation. Genomic DNA was digested with PstI and MspI following the GBS protocol of [10]. After ligation of barcode adapters and size selection (200–300 bp), pooled libraries were sequenced on an Illumina NovaSeq 6000 platform (Illumina Inc., San Diego, CA, USA) by Novogene Bioinformatics Technology Co., Ltd., (Beijing, China) using paired-end 150 bp reads. Raw sequence reads were assessed using FastQC v0.11.9 [24], trimmed with Trimmomatic v0.39 [25], and demultiplexed prior to downstream analyses. SNPs were identified de novo using Stacks v2.0 [26]. The resulting SNP dataset was used for phylogenetic reconstruction using the ML method implemented in RAxML v8.0 [27]. Branch support was assessed using 1000 bootstrap replicates. The raw GBS sequencing data have been deposited in the Genome Sequence Archive (GSA), National Genomics Data Center (NGDC), China National Center for Bioinformation (CNCB), under accession number CRA045255.

### 4.2. trnK Intron and ITS Amplification, Sequencing, and Phylogenetic Analysis

Twenty *Nepenthes* taxa were analyzed using plastid trnK intron and nuclear ribosomal ITS sequence data. Multiple accessions of *N*. *mirabilis*, *N*. *mirabilis* var. *globosa*, and *N*. *kampotiana* were included to assess sequence consistency within taxa. Genomic DNA was extracted using the modified CTAB protocol described in Section 4.1. Detailed information on sampled taxa, voucher specimens, collection localities, and sequence accession numbers is provided in Supplementary Table S7.

For trnK intron analysis, twenty-three newly generated sequences were obtained from Thai *Nepenthes* taxa. The trnK intron region was amplified using the primer pairs 2-trnK-3914F/Nep16-1270R and Nep2-1060F/16-trnK-2R following Meimberg et al. [15] (Table S8). PCR amplification consisted of an initial denaturation at 94 °C for 2 min, followed by 40 cycles of 94 °C for 1 min, 54 °C for 1 min, and 72 °C for 2 min, with a final extension at 72 °C for 10 min. Purified PCR products were sequenced by Pacific Science Co., Ltd. (Bangkok, Thailand). The newly generated trnK intron sequences were deposited in GenBase (National Genomics Data Center, China National Center for Bioinformation) under accession numbers C_AA462228.1–C_AA462250.1.

Sequence alignment was performed using MUSCLE implemented in MEGA v6.0 [28]. Newly generated sequences were analyzed together with representative *Nepenthes* trnK intron sequences retrieved from GenBank, including taxa reported by Meimberg et al. [15]. *A. abbreviatus* and *T. peltatum* were used as outgroup taxa. The Kimura two-parameter (K2P) model, identified as the best-fit substitution model in MEGA v6.0 [28], was selected for phylogenetic reconstruction. ML analysis was performed with 1000 bootstrap replicates to assess branch support.

For ITS analysis, twenty-three newly generated sequences were obtained from Thai *Nepenthes* taxa and analyzed together with representative ITS sequences retrieved from GenBank, including taxa reported by Meimberg et al. [7]. The ITS region was amplified using the primer pair AITS1-F and AITS4-R following Meimberg et al. [7] (Table S8). Each 25 μL PCR reaction contained 10 ng genomic DNA, 1× PCR buffer, 2.0 mM MgCl_2_, 0.2 mM dNTPs, 0.4 μM of each primer, and 1 U Taq DNA polymerase. PCR amplification consisted of an initial denaturation at 96 °C for 90 s, followed by 40 cycles of 96 °C for 20 s, 57 °C for 40 s, and 72 °C for 40 s, with a final extension at 72 °C for 10 min. Purified PCR products were sequenced bidirectionally by Pacific Science Co., Ltd. (Bangkok, Thailand). The newly generated ITS sequences were deposited in GenBase (National Genomics Data Center, China National Center for Bioinformation) under accession numbers C_AA474089.1–C_AA474111.1.

Sequence alignment was performed using MUSCLE implemented in MEGA v6.0 [28]. *Dionaea muscipula* was selected as the outgroup taxon. The best-fit substitution model was determined in MEGA v6.0, and the Kimura two-parameter (K2P) model was selected for phylogenetic reconstruction. ML analysis was conducted with 1000 bootstrap replicates to evaluate branch support.

### 4.3. Genetic Analysis of N. mirabilis Using AFLP Markers and ITS Sequences

Thirty-three *N*. *mirabilis* accessions representing populations distributed throughout southern Thailand were included in the AFLP analysis (Table S9). Genomic DNA was extracted using the modified CTAB method described in Section 4.1.

AFLP analysis was performed following a modified protocol of [12]. Genomic DNA was digested with EcoRI and MseI restriction enzymes overnight, followed by ligation of corresponding adapters at 37 °C. Pre-selective amplification was conducted using primer combinations containing one selective nucleotide (ER+1/MS+1) under the following conditions: 20 cycles of 95 °C for 30 s, 56 °C for 30 s, and 72 °C for 1 min. Selective amplification employed combinations of ER+2/3 and MS+3 primers (Table S10) using a two-step PCR program consisting of 10 touchdown cycles with annealing temperatures decreasing from 65 °C to 56 °C, followed by 30 cycles at 56 °C. Amplified fragments were separated on 5% denaturing polyacrylamide gels and visualized by silver staining. Only clear and reproducible bands were scored and included in the analysis, with band presence and absence recorded as 1 and 0, respectively, to generate a binary data matrix. Genetic similarity coefficients were calculated using Jaccard’s coefficient [29], and cluster analysis was performed using the unweighted pair-group method with arithmetic mean (UPGMA) implemented in NTSYSpc v2.21 (Exeter Software, Setauket, NY, USA) [30].

For ITS analysis, thirty-three newly generated ITS sequences were obtained and analyzed together with reference *Nepenthes* ITS sequences retrieved from GenBank, including representative taxa reported by [7]. The ITS region was amplified using the primer pair AITS1-F and AITS4-R following [7] (Table S8). Each 25 μL PCR reaction contained 10 ng genomic DNA, 1× PCR buffer, 2.0 mM MgCl_2_, 0.2 mM dNTPs, 0.4 μM of each primer, and 1 U Taq DNA polymerase. PCR amplification consisted of an initial denaturation at 96 °C for 90 s, followed by 40 cycles of denaturation at 96 °C for 20 s, annealing at 57 °C for 40 s, and extension at 72 °C for 40 s, with a final extension at 72 °C for 10 min. Purified PCR products were sequenced bidirectionally by Pacific Science Co., Ltd. (Bangkok, Thailand). The newly generated ITS sequences have been deposited in GenBase, National Genomics Data Center (NGDC), China National Center for Bioinformation (CNCB), under accession numbers C_AA474152.1–C_AA474184.1. Sequence alignment was performed using MUSCLE implemented in MEGA v6.0 [28]. *D. muscipula* was selected as the outgroup for phylogenetic analysis. The best-fit nucleotide substitution model was determined using MEGA v6.01, and the Kimura two-parameter (K2P) model was selected for phylogenetic reconstruction. ML analysis was conducted with 1000 bootstrap replicates to assess branch support.

## 5. Conclusions

Integrating GBS-derived SNPs, trnK intron sequences, ITS sequences, and AFLP markers provided a robust molecular framework for assessing phylogenetic relationships among Thai *Nepenthes* taxa. The combined datasets recovered largely congruent phylogenetic patterns and improved resolution among several closely related taxa. Across all datasets, *N*. *mirabilis* var. *globosa* was consistently recovered within the broader *N*. *mirabilis* complex and exhibited limited genetic differentiation from sampled *N*. *mirabilis* accessions. These findings provide new molecular evidence for understanding phylogenetic relationships within Thai *Nepenthe*s and establish a foundation for future taxonomic, evolutionary, and conservation research.

## Figures and Tables

**Table 1:** Summary of AFLP and genetic diversity statistics among 33 *Nepenthes mirabilis* accessions.

Primer Pairs	Total No. of Bands	PB	MB	P	M	PIC
ER-AC/MS-CAC	67	28	39	41.79	58.21	0.14
ER-AG/MS-CAC	71	28	43	39.44	60.56	0.19
ER-AGA/MS-CAC	72	37	35	51.39	48.61	0.23
ER-ACC/MS-CAC	71	42	29	59.15	40.85	0.16
ER-AAG/MS-CAC	78	31	47	39.74	60.26	0.13
ER-AAC/MS-CAC	66	31	35	46.97	53.03	0.21
ER-ACA/MS-CAC	67	30	37	44.78	55.22	0.12
ER-AGC/MS-CAC	76	22	54	28.95	71.05	0.09
ER-ATG/MS-CAC	74	28	46	37.84	62.16	0.16
ER-AGG/MS-CAC	68	31	37	45.59	54.41	0.21
ER-AGG/MS-CAT	32	15	17	46.88	53.13	0.27
Total	742	323	419	-	-	1.91
Mean	67.45	29.36	38.09	43.86	56.14	0.17

PB, polymorphic bands; MB, monomorphic bands; P, percentage of polymorphic bands; M, percentage of monomorphic bands; PIC, polymorphism information content.

## Data Availability

The raw genotyping-by-sequencing (GBS) data and nucleotide sequences generated for the genetic and phylogenetic analyses of Thai *Nepenthes* taxa have been deposited in the National Genomics Data Center (NGDC), China National Center for Bioinformation (CNCB), Beijing Institute of Genomics, Chinese Academy of Sciences. The GBS data are available in the Genome Sequence Archive (GSA) under accession number CRA045255, while the chloroplast trnK intron and nuclear ITS sequences are available in GenBase under accession numbers C_AA462228.1–C_AA462250.1 and C_AA474089.1–C_AA474111.1, respectively. The nuclear ITS sequences generated for the genetic analysis of the *Nepenthes mirabilis* complex are available in GenBase under accession numbers C_AA474152.1–C_AA474184.1. All datasets are scheduled for public release on 6 August 2026.

## Supplementary Materials

The following supporting information can be downloaded at: https://www.mdpi.com/article/10.3390/plants15142238/s1, Figure S1: Visualization of nucleotide variation in the trnK intron region among 90 *Nepenthes* taxa based on MUSCLE sequence alignment. Different colors indicate nucleotide variation, whereas white gaps represent alignment gaps and missing sequence data. *Ancistrocladus abbreviatus* and *Triphyophyllum peltatum* were included as outgroup taxa; Table S1: Sequencing quality statistics of Thai *Nepenthes* samples generated by genotyping-by-sequencing (GBS); Table S2: Summary statistics of de novo assembly generated from GBS sequencing data of Thai *Nepenthes*; Table S3: Summary of mapping statistics, sequencing depth, genome coverage, and tag distribution for each *Nepenthes* sample used in GBS analysis; Table S4: Summary statistics of SNP variation identified from genotyping-by-sequencing (GBS) data in Thai *Nepenthes*, including transition/transversion ratios and heterozygosity rates; Table S5: Pairwise genetic similarity coefficients among 33 *Nepenthes mirabilis* accessions calculated from AFLP data using Jaccard’s similarity coefficient. Similarity values range from 0 (no similarity) to 1 (complete similarity); Table S6: *Nepenthes* taxa, collection localities, and geographic coordinates used for genotyping-by-sequencing (GBS) analysis; Table S7: *Nepenthes* taxa, collection localities, geographic coordinates, and sequence accession numbers used in the trnK intron and ITS phylogenetic analyses; Table S8: Primers used for amplification of the trnK intron and ITS regions, including nucleotide sequences and melting temperatures (Tm); Table S9: Sampling localities and geographic coordinates of 33 *Nepenthes mirabilis* accessions used for AFLP and ITS analyses; Table S10: Pre-selective and selective AFLP primer sequences used for genetic analysis of Thai *Nepenthes mirabilis*.

## Abbreviations

The following abbreviations are used in this manuscript:

AFLP	Amplified Fragment Length Polymorphism
CTAB	Cetyltrimethylammonium bromide
DNA	Deoxyribonucleic acid
GBS	Genotyping-by-Sequencing
ITS	Internal Transcribed Spacer
M	Percentage of monomorphic bands
matK	Maturase K
MB	Monomorphic bands
NCBI	National Center for Biotechnology Information
P	percentage of polymorphic bands
PB	Polymorphic bands
PCR	Polymerase Chain Reaction
PIC	Polymorphism information content
rbcL	Ribulose-1,5-bisphosphate carboxylase/oxygenase large subunit
SNPs	Single Nucleotide Polymorphisms
trnH–psbA	tRNA-His (trnH)—Photosystem II protein D1 (psbA) intergenic spacer
trnK	Chloroplast transfer RNA gene for Lysine
UPGMA	Unweighted Pair Group Method with Arithmetic Mean
