# Supplementary material for: Phylogenetic Relationships and Genetic Diversity of Thai Nepenthes (Nepenthaceae) Revealed by Integrative Molecular Analyses
Source: Plants (Basel). 2026 Jul 22;15(14):2238. doi: 10.3390/plants15142238 (PMC13417141; doi:10.3390/plants15142238)
Supplement: Supplementary file 1 [file plants-15-02238-s001.zip › plants-4369981-supplementary.pdf]

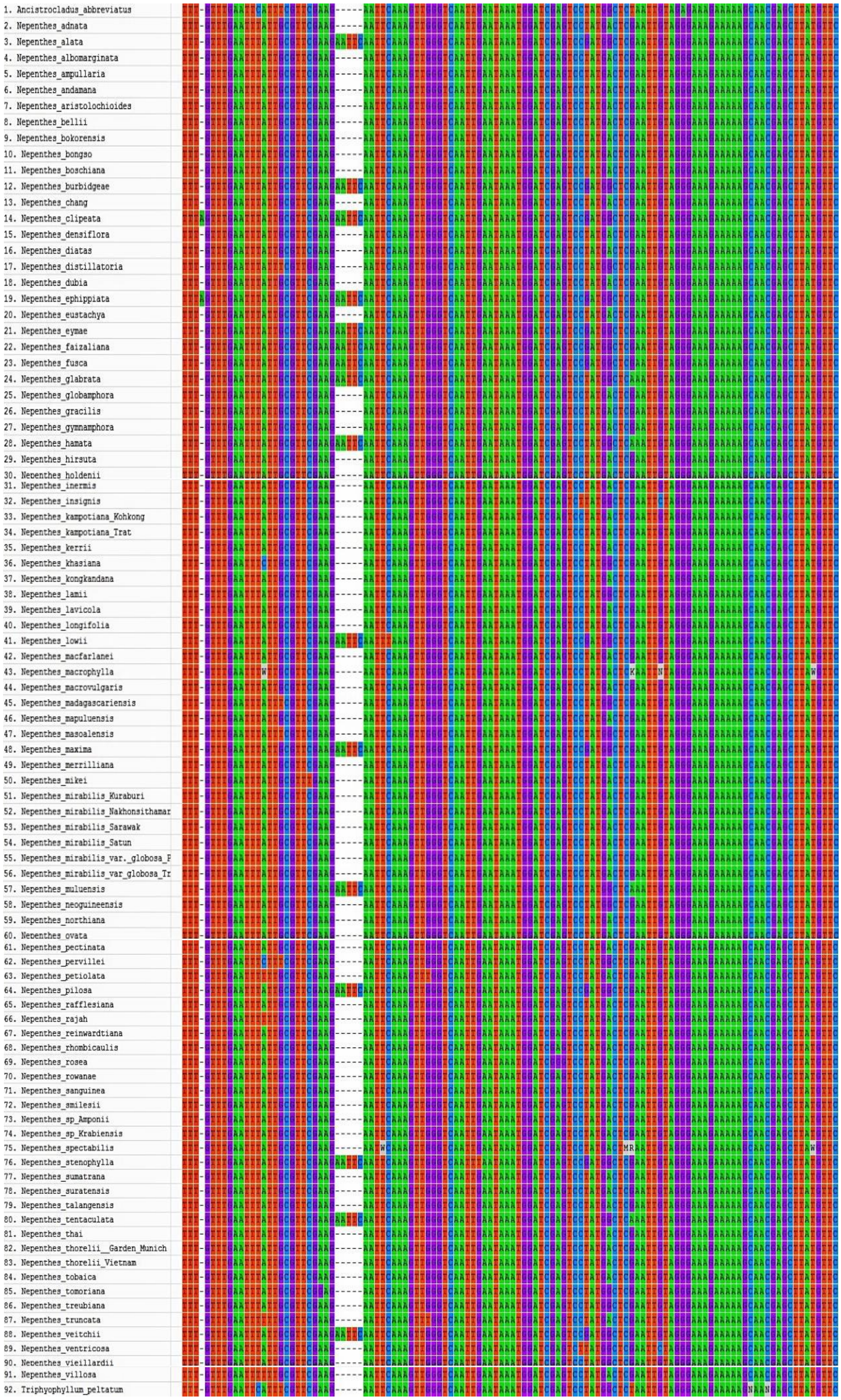

Figure S1. Visualization of nucleotide variation in the plastid trnK intron region among 90 *Nepenthes* taxa based on MUSCLE sequence alignment. Different colors indicate nucleotide variation, whereas white gaps represent alignment gaps and missing sequence data. *Ancistrocladus abbreviatus* and *Triphyophyllum peltatum* were included as outgroup taxa.

Table S1. Sequencing quality statistics of Thai *Nepenthes* samples generated by genotyping– by–sequencing (GBS).

Sample	Raw bases (bp)	Clean bases (bp)	Effective rate (%)	Error rate (%)	Q20 (%)	Q30 (%)	GC content (%)
Ampullaria1	719252928	718834830	99.94	0.03	98.00	94	38.05
Ampullaria3	648559872	648152898	99.94	0.03	97.73	93.3	38.11
Ampullaria5	747173664	746671394	99.93	0.03	97.69	93.22	38.5
Andamana1	765150912	764722438	99.94	0.03	97.53	92.62	38.48
Andamana2	650898720	650233728	99.9	0.03	97.60	93.03	38.7
Bracteosa1	643927104	643492598	99.93	0.03	97.74	93.25	38.56
Bracteosa3	748054656	747559602	99.93	0.03	98.03	94.15	38.45
Bracteosa4	623670912	623118432	99.91	0.03	97.52	92.58	38.72
Chang2	918836352	918305876	99.94	0.03	96.10	89.05	39.95
Chang3	1845787968	1844296394	99.92	0.04	94.72	86.42	40.13
Chang4	788433984	788052310	99.95	0.04	94.13	84.88	40.07
GlobosaTra3	614829312	614324562	99.92	0.03	97.84	93.66	38.13
GlobosaTra4	664738848	664360472	99.94	0.03	97.79	93.45	37.88
GlobosaTra5	704124576	703768134	99.95	0.03	97.63	92.98	36.89
Gracilis	755894880	755695200	99.97	0.03	97.73	93.28	37.28
Hirtella1	730786752	730322276	99.94	0.03	97.92	93.84	38.44
Hirtella2	669426624	668933382	99.93	0.03	97.63	92.95	38.92
Hirtella3	735005088	734626496	99.95	0.03	97.81	93.47	38.16
Kampotiana	728630496	728003042	99.91	0.03	97.79	93.38	38.83
Kerrii1	803105856	802369766	99.91	0.03	97.64	92.97	38.28
Kerrii2	761035392	760344724	99.91	0.03	97.75	93.32	38.12
Kongkandana3	679140576	678661676	99.93	0.03	97.60	92.81	38.21
Kongkandana4	707609376	707049890	99.92	0.03	97.54	92.67	38.43
Krabiensis1	619437888	618838900	99.9	0.03	97.91	93.82	38.59
Krabiensis3	655106112	654520352	99.91	0.03	97.82	93.47	38.66
Krabiensis6	703602432	703109848	99.93	0.03	97.29	92.03	38.67
Mirabilis3	716833440	716336122	99.93	0.03	97.73	93.31	38.12
Mirabilis4	775894176	775362114	99.93	0.03	97.48	92.59	38.07
MirabilisGP1	813172608	812738912	99.95	0.03	97.78	93.46	38.11
MirabilisGP3	754291584	753855180	99.94	0.03	97.37	92.3	37.79
Rafflesiana1	801809568	801358212	99.94	0.03	97.49	92.64	38.12
Rafflesiana2	881331264	880960926	99.96	0.03	97.69	93.24	38.09
Rafflesiana3	813220128	812703036	99.94	0.03	97.63	93.07	38.55
Rosea	704180736	703599562	99.92	0.03	97.80	93.38	37.89
Sanguinea	623863872	623014698	99.86	0.03	97.83	93.53	38.19
Smilesii1	764172288	763681554	99.94	0.03	97.69	93.16	37.99
Smilesii2	765742752	765164690	99.92	0.03	97.98	94	38.62
Smilesii3	670767840	670267582	99.93	0.03	97.72	93.23	38.71
Smilesii4	825466464	824855130	99.93	0.03	97.84	93.53	38.21
Suratensis1	718038720	717520022	99.93	0.03	97.75	93.31	38.02
Suratensis3	793586592	793260120	99.96	0.03	96.66	90.42	38.34
Thai3	697281120	696600198	99.9	0.03	97.81	93.44	38.23
Thai4	742231008	741604024	99.92	0.03	97.38	92.21	38.78

Table S1. Sequencing quality statistics of Thai *Nepenthes* samples generated by genotyping– by–sequencing (GBS). (Count.)

Sample	Raw bases (bp)	Clean bases (bp)	Effective rate (%)	Error rate (%)	Q20 (%)	Q30 (%)	GC content (%)
Thorelii2	890913600	890409360	99.94	0.03	97.74	93.23	38.41
Thorelii3	782442432	781962414	99.94	0.03	97.49	92.53	38.94
Unknow1	518988960	518663282	99.94	0.03	97.55	92.71	38.91
Unknow2	710190144	709765504	99.94	0.03	98.06	94.16	38.3

Raw base (bp): The result of raw data calculated by the number and length of base sequences (in bp units).
Clean base (bp): Total number of high-quality bases retained after quality filtering and adapter trimming.
Effective rate (%)**:** The ratio of clean data to raw data.
Error rate (%): Estimated sequencing error rate across all bases.
Q20 and Q30 (%): The percentage of bases with Phred scores higher than 20 and 30 among all bases. The Phred value (Qphred = -10log10(e)) of sequencing error rate (e) indicates the quality of base sequences obtained from library construction.
GC Content (%): The percentage of G and C bases among all bases.

Table S2. Summary statistics of de novo assembly generated from GBS sequencing data of Thai *Nepenthes*.

Seq number	Total length	GC content (%)	Gap rate (%)	N50 length	N90 length
742845	180,450,637	36.82	0.00	256	204

Seq number: Total number of assembled contigs.
Total length: Combined length of all assembled contigs.
GC content (%): Percentage of guanine (G) and cytosine (C) nucleotides in the assembled sequences.
Gap rate (%): Proportion of unresolved bases or gaps in the assembled sequences.
N50 length: Contig length at which 50% of the total assembly length is contained in contigs of this length or longer.
N90 length: Contig length at which 90% of the total assembly length is contained in contigs of this length or longer.

Table S3. Summary of mapping statistics, sequencing depth, genome coverage, and tag distribution for each *Nepenthes* sample used in GBS analysis.

Sample	Mapped reads	Total reads	Tag number	Tag4 number	Mapping rate (%)	Average depth (X)	Coverage at least 1X (%)	Coverage at least 4X (%)
Thai4	4333138	5154142	555965	281909	84.07	10.73	30.44	19.83
Sanguinea	3489626	4332180	487136	200988	80.55	8.98	26.38	14.22
Rafflesiana2	3442833	6120054	454863	231371	56.25	10.93	23.31	15.3
Ampullaria3	2962154	4503668	443374	198953	65.77	9.65	22.3	13.43
Kongkandana3	4075424	4716038	608583	282484	86.42	9.1	34.06	20.78
Rosea	3956357	4889918	606347	261086	80.91	8.76	33.76	19.28
Ampullaria1	3438230	4994574	417657	214445	68.84	11.66	21.51	14.14
Andamana2	3551724	4519914	689644	268243	78.58	7.3	36.6	20.06
Ampullaria5	3520755	5188436	462266	218190	67.86	11.11	23.07	14.56
Kongkandana4	4329044	4913722	614208	291512	88.1	9.29	35.48	21.63
Rafflesiana1	3981396	5567858	475625	246523	71.51	12.39	23.77	16.07
Andamana1	4700628	5313324	589523	304316	88.47	10.72	33.47	21.82
Suratensis3	4890016	5510728	593486	311080	88.74	11.15	33.67	22.43
Smilesii1	4786618	5732082	492066	254448	83.51	12.5	27.52	17.74
Rafflesiana3	3896945	5647036	488054	241701	69.01	11.85	24.32	15.96
Smilesii2	4484726	5306498	634195	273376	84.51	10.37	32.26	19.33
Unknown1	2452231	3603894	488321	184358	68.04	6.97	26.26	13.55
Chang2	6004259	6380194	684040	353623	94.11	11.73	39.3	25.48
Suratensis1	4305547	4986100	757560	289983	86.35	8.12	40.06	22.16
Mirabilis3	3706186	4977734	472094	247212	74.46	11.03	25.38	17.1
Unknown2	3527085	4931592	532196	235996	71.52	9.28	28.29	16.72
Mirabilis4	4089084	5387834	477130	256627	75.89	12.22	25.31	17.61
GlobosaTra5	3734628	4889508	490455	238715	76.38	10.96	25.48	16.58
Bracteosa4	3742628	4330828	635674	272131	86.42	8.17	34.87	20.29
Hirtella3	4536473	5103960	596072	294541	88.88	9.86	34.73	21.74
Gracilis	3291684	5248992	393453	199902	62.71	11.79	20.23	13.11
Kerrii	4677151	5576824	566430	278341	83.87	10.81	32.58	20.07
Krabiensis1	3613678	4301492	656759	273447	84.01	7.7	35.74	20.63
Bracteosa1	3927962	4471478	569205	263203	87.84	9.03	32.6	19.57
Krabiensis6	4204480	4885904	644037	298814	86.05	9.07	35.49	22.11
Hirtella2	3975675	4648562	611423	283022	85.52	8.85	34.44	21
MirabilisGP1	4388405	5646712	459898	267398	77.72	13.18	25.42	18.47
Chang4	5156349	5474818	582266	303621	94.18	12.09	32.6	21.77
Smilesii3	3973895	4657870	599514	262397	85.32	9.38	31.98	19.03
Hirtella1	4416062	5074698	601813	287052	87.02	9.63	34.41	21.31
Thai3	4084240	4841964	633701	283482	84.35	8.91	34.52	20.67
MirabilisGP3	4165234	5237852	506315	285344	79.52	10.94	29.35	20.21
Thorelii2	5091123	6186572	552606	301823	82.29	12.43	31.19	21.28

Table S3. Summary of mapping statistics, sequencing depth, genome coverage, and tag distribution for each *Nepenthes* sample used in GBS analysis. (Cont.)

Sample	Mapped reads	Total reads	Tag number	Tag4 number	Mapping rate (%)	Average depth (X)	Coverage at least 1X (%)	Coverage at least 4X (%)
Kampotiana	4068772	5059668	604584	269419	80.42	9.16	33.44	19.48
Thorelii3	4462959	5433358	527717	282879	82.14	11.27	30.19	20.14
GlobosaTra3	3068172	4269420	500794	224184	71.86	8.72	26.5	16.13
Bracteosa3	4494053	5194576	615320	305995	86.51	9.87	34.91	22.39
Krabiensis3	3915225	4549116	682057	287360	86.07	7.89	38.01	21.88
GlobosaTra4	3547937	4615976	451465	235480	76.86	10.75	24.7	16.47
Chang3	11642873	12817046	1020544	481382	90.84	17.87	49	33.67
Kerrii1	4543075	5284678	659164	306531	85.97	9.64	35.7	22.48
Smilesii4	4530581	5317370	544272	278567	85.2	11.35	30.02	19.67

Sample: Sample names.
Mapped reads: Number of clean reads mapped to the de novo assembled reference sequences.
Total reads: Total number of high-quality reads retained after quality filtering.
Tag number: Total number of unique tags (restriction enzyme fragments) identified in each sample.
Tag4 number: Number of tags with sequencing depth greater than 4.
Mapping rate (%): Percentage of clean reads successfully mapped to the de novo assembly.
Average depth (X): The average sequencing depth across mapped sites, calculated as the total number of bases in mapped reads divided by the length of the covered de novo assembly.
Coverage at least 1X (%): Percentage of assembled sequences covered by at least one read.
Coverage at least 4X (%): Percentage of assembled sequences covered by four or more reads.

Table S4. Summary statistics of SNP variation identified from genotyping–by–sequencing (GBS) data in Thai *Nepenthes*, including transition/transversion ratios and heterozygosity rates.

Sample	ts	tv	ts/tv	Het rate (‰)	Total
Thai4	260,936	125,570	2.077	0.727	386,506
Sanguinea	249,622	123,691	2.018	0.816	373,313
Rafflesiana2	394,671	164,511	2.399	0.888	559,182
Ampullaria3	399,975	159,802	2.502	0.725	559,777
Kongkandana3	147,970	84,115	1.759	0.465	232,085
Rosea	152,633	89,093	1.713	0.526	241,726
Ampullaria1	393,774	159,509	2.468	0.604	553,283
Andamana2	252,166	133,318	1.891	1.104	385,484
Ampullaria5	432,189	173,276	2.494	0.751	605,465
Kongkandana4	153,872	87,343	1.761	0.514	241,215
Rafflesiana1	415,969	172,666	2.409	0.934	588,635
Andamana1	177,579	98,552	1.801	0.580	276,131
Suratensis3	161,232	92,216	1.748	0.501	253,448
Smilesii1	184,356	94,085	1.959	0.433	278,441
Rafflesiana3	423,035	174,899	2.418	0.985	597,934
Smilesii2	228,611	117,103	1.952	0.687	345,714
Unknown1	253,933	118,612	2.140	0.768	372,545
Chang2	133,989	91,960	1.457	0.611	225,949
Suratensis1	198,994	107,841	1.845	0.803	306,835
Mirabilis3	287,179	127,813	2.246	0.717	414,992
Unknown2	303,206	138,352	2.191	0.859	441,558
Mirabilis4	295,246	131,404	2.246	0.727	426,650
GlobosaTra5	270,767	122,382	2.212	0.728	393,149
Bracteosa4	206,468	115,346	1.789	0.832	321,814
Hirtella3	147,206	85,638	1.718	0.482	232,844
Gracilis	371,365	157,601	2.356	0.677	528,966
Kerrii	134,920	78,687	1.714	0.383	213,607
Krabiensis1	226,577	121,475	1.865	0.908	348,052
Bracteosa1	172,488	96,861	1.780	0.596	269,349
Krabiensis6	201,560	112,111	1.797	0.739	313,671
Hirtella2	180,280	102,355	1.761	0.679	282,635
MirabilisGP1	249,966	118,204	2.114	0.664	368,170
Chang4	107,416	74,863	1.434	0.374	182,279
Smilesii3	252,102	128,321	1.964	0.842	380,423
Hirtella1	182,506	99,246	1.838	0.629	281,752
Thai3	220,976	110,955	1.991	0.686	331,931
MirabilisGP3	184,762	97,942	1.886	0.624	282,704
Thorelii2	183,603	98,951	1.855	0.494	282,554

Table S4. Summary statistics of SNP variation identified from genotyping–by–sequencing (GBS) data in Thai *Nepenthes*, including transition/transversion ratios and heterozygosity rates. (Cont.)

Sample	ts	tv	ts/tv	Het rate (‰)	Total
Kampotiana	217,994	112,433	1.938	0.948	330,427
Thorelii3	174,095	94,262	1.846	0.478	268,357
GlobosaTra3	276,376	123,491	2.238	0.769	399,867
Bracteosa3	191,409	106,989	1.789	0.662	298,398
Krabiensis3	200,762	112,223	1.788	0.832	312,985
GlobosaTra4	264,434	119,781	2.207	0.707	384,215
Chang3	305,045	179,271	1.701	1.559	484,316
Kerrii1	200,504	111,669	1.795	0.686	312,173
Smilesii4	208,563	107,147	1.946	0.513	315,710

Sample: Sample name.
Ts (Transition): A nucleotide substitution between two purines (A ↔ G) or two pyrimidines (C ↔ T).
Tv (Transversion): A nucleotide substitution between a purine and a pyrimidine (e.g., A ↔ C, A ↔ T, G ↔ C, or G ↔ T).
Ts/Tv: Ratio of transition mutations to transversion mutations.
Het rate (‰): Genome-wide heterozygosity estimated as the proportion of heterozygous SNPs relative to the total number of analyzed sites.
Total: Total number of SNPs identified in each sample.

Table S5. Pairwise genetic similarity coefficients among 33 *Nepenthes mirabilis* accessions calculated from AFLP data using Jaccard's similarity coefficient. Similarity values range from 0 (no similarity) to 1 (complete similarity).

	S1	S2	S3	S4	S5	S6	S7	S8	S9	S10	S11	S12	S13	S14	S15	S16	S17	S18	S19	S20	S21	S22	S23	S24	S25	S26	S27	S28	S29	S30	S31	S32	S33
**S1**	1.000																																
**S2**	0.938	1.000																															
**S3**	0.922	0.934	1.000																														
**S4**	0.926	0.942	0.934	1.000																													
**S5**	0.919	0.925	0.924	0.937	1.000																												
**S6**	0.927	0.936	0.940	0.939	0.926	1.000																											
**S7**	0.910	0.916	0.938	0.934	0.935	0.932	1.000																										
**S8**	0.917	0.938	0.951	0.947	0.939	0.944	0.936	1.000																									
**S9**	0.939	0.958	0.936	0.220	0.939	0.934	0.962	0.920	1.000																								
**S10**	0.924	0.929	0.936	0.929	0.945	0.927	0.110	0.926	0.917	1.000																							
**S11**	0.004	0.914	0.913	0.908	0.915	0.886	0.916	0.893	0.926	0.900	1.000																						
**S12**	0.915	0.915	0.904	0.928	0.885	0.920	0.905	0.916	0.912	0.944	0.912	1.000																					
**S13**	0.826	0.840	0.844	0.808	0.842	0.825	0.848	0.839	0.845	0.828	0.849	0.823	1.000																				
**S14**	0.882	0.886	0.891	0.892	0.894	0.893	0.896	0.853	0.904	0.871	0.867	0.813	0.890	1.000																			
**S15**	0.926	0.914	0.927	0.906	0.914	0.919	0.901	0.915	0.911	0.905	0.836	0.924	0.894	0.916	1.000																		
**S16**	0.934	0.936	0.926	0.926	0.902	0.934	0.904	0.923	0.832	0.943	0.890	0.920	0.904	0.941	0.915	1.000																	
**S17**	0.933	0.933	0.900	0.956	0.905	0.927	0.828	0.944	0.903	0.945	0.923	0.945	0.931	0.953	0.944	0.938	1.000																
**S18**	0.913	0.945	0.921	0.920	0.840	0.937	0.898	0.932	0.933	0.950	0.915	0.943	0.945	0.939	0.956	0.935	0.946	1.000															
**S19**	0.902	0.927	0.833	0.935	0.897	0.948	0.926	0.943	0.925	0.930	0.944	0.935	0.957	0.933	0.945	0.947	0.909	0.947	1.000														
**S20**	0.829	0.939	0.898	0.911	0.907	0.932	0.906	0.934	0.928	0.933	0.950	0.914	0.920	0.931	0.893	0.931	0.938	0.909	0.911	1.000													
**S21**	0.887	0.920	0.933	0.935	0.907	0.920	0.931	0.916	0.938	0.923	0.932	0.943	0.922	0.922	0.937	0.938	0.924	0.920	0.928	0.837	0.934	1.000											
**S22**	0.907	0.941	0.887	0.911	0.945	0.916	0.929	0.912	0.935	0.925	0.928	0.934	0.933	0.938	0.906	0.917	0.843	0.925	0.937	0.881	0.920	1.000											
**S23**	0.913	0.939	0.921	0.935	0.923	0.934	0.922	0.922	0.930	0.935	0.929	0.911	0.925	0.844	0.922	0.922	0.884	0.920	0.954	0.907	0.941	0.903	1.000										
**S24**	0.940	0.936	0.936	0.936	0.907	0.944	0.925	0.908	0.925	0.936	0.842	0.941	0.944	0.895	0.925	0.927	0.912	0.943	0.905	0.929	0.936	0.927	0.932	1.000									
**S25**	0.006	0.878	0.912	0.908	0.885	0.901	0.898	0.826	0.906	0.910	0.854	0.907	0.895	0.897	0.914	0.884	0.897	0.901	0.903	0.891	0.917	0.902	0.913	0.904	1.000								
**S26**	0.765	0.762	0.753	0.774	0.750	0.761	0.777	0.739	0.741	0.759	0.749	0.765	0.745	0.755	0.777	0.760	0.764	0.776	0.765	0.758	0.748	0.738	0.774	0.763	0.761	1.000							
**S27**	0.910	0.840	0.921	0.924	0.853	0.914	0.910	0.904	0.920	0.889	0.912	0.907	0.907	0.901	0.915	0.920	0.908	0.902	0.889	0.913	0.901	0.918	0.777	0.926	0.900	0.905	1.000						
**S28**	0.912	0.883	0.919	0.915	0.908	0.925	0.899	0.917	0.912	0.935	0.906	0.914	0.928	0.910	0.927	0.879	0.924	0.886	0.915	0.767	0.937	0.896	0.901	0.906	0.918	0.828	0.921	1.000					
**S29**	0.927	0.917	0.934	0.902	0.922	0.921	0.941	0.914	0.928	0.937	0.916	0.936	0.894	0.938	0.901	0.924	0.770	0.949	0.905	0.896	0.914	0.924	0.852	0.924	0.948	0.930	0.889	0.930	1.000				
**S30**	0.919	0.906	0.925	0.910	0.916	0.921	0.895	0.915	0.873	0.926	0.900	0.908	0.782	0.921	0.909	0.886	0.897	0.917	0.849	0.917	0.941	0.912	0.874	0.903	0.953	0.923	0.905	0.921	0.890	1.000			
**S31**	0.917	0.931	0.904	0.916	0.879	0.938	0.898	0.923	0.775	0.922	0.905	0.904	0.903	0.909	0.841	0.918	0.902	0.924	0.877	0.907	0.914	0.915	0.906	0.925	0.891	0.910	0.900	0.921	0.923	0.914	1.000		
**S32**	0.791	0.927	0.895	0.912	0.783	0.920	0.913	0.901	0.917	0.912	0.838	0.821	0.937	0.935	0.869	0.901	0.946	0.915	0.906	0.925	0.888	0.950	0.912	0.910	0.918	0.914	0.925	0.920	0.919	0.902	0.916	1.000	
**S33**	0.791	0.897	0.885	0.873	0.900	0.895	0.839	0.903	0.883	0.898	0.863	0.890	0.899	0.895	0.891	0.897	0.863	0.901	0.884	0.898	0.900	0.883	0.897	0.902	0.896	0.879	0.890	0.899	0.871	0.901	0.876	0.892	1.000

Table S6. *Nepenthes* taxa, collection localities, and geographic coordinates used for genotyping–by–sequencing (GBS) analysis.

No.	Sample	Sources	Location
1	*N. mirabilis* (Lour.) Druce	Nakhon Si Thammarat, Thailand	8.3475334N, 99.7125974E
2	*N. mirabilis* (Lour.) Druce	Satun, Thailand	7.0338056N, 100.0282917E
3	*N. mirabilis* var. *globosa* M.Catal.	Trang, Thailand	7.5153462N, 99.5783642E
4	*N. ampullaria* Jack	Songkhla, Thailand	7.0281542N, 100.3743426E
5	*N. gracilis* Korth.	Phatthalung, Thailand	7.2729117N, 100.1920231E
6	*N. rosea* M.Catal. & Kruetr.	Krabi, Thailand	8.0878785N, 98.7756099E
7	*N. kongkandana* M.Catal. & Kruetr.	Songkhla, Thailand	6.8845421N, 100.9360950E
8	*N. andamana* M.Catal	Phang–Nga, Thailand	8.9012219N, 98.2701050E
9	*N. suratensis* M.Catal	Suratthani, Thailand	9.1897309N, 99.5407791E
10	*N. kerrii* M.Catal. & Kruetr.	Satun, Thailand	6.5158120N, 99.3110529E
11	*N. hirtella* Nuanlaong & Suran. *sp. nov.*	Krabi, Thailand	7.8407692N, 99.2763659E
12	*N. krabiensis* Nuanlaong, Onsanit, Chusangr. & Suraninpong.	Krabi, Thailand	7.8992101N, 99.3032899E
13	*N. bracteosa* Suran. & Nuanlaong *sp. nov.*	Nakhon Si Thammarat, Thailand	8.2684420N, 99.6706821E
14	*N. chang* M.Catal	Trat, Thailand	12.0251375N, 102.3411471E
15	*N. kampotiana* Lecomte	Trat, Thailand	11.9053270N, 102.7988802E
16	*N. thai* Cheek	Narathiwat, Thailand	5.8268963N, 101.6901186E
17	*N. sanguinea* Lindl.	Yala, Thailand	5.9153922N, 101.3068112E
18	*N. smilesii* Hemsl.	Loei, Thailand	16.8608208N, 101.7505850E
19	*N. rafflesiana* Jack	Johor Bahru, Malaysia	2.52990887N, 103.41185691E
20	*N. thorelii* Lecomte	Tay Ninh, Vietnam	11.6386368N, 105.9044199E
21	Unknown	Suratthani, Thailand	8.7791088N, 98.6821604E

Table S7. *Nepenthes* taxa, collection localities, geographic coordinates, and sequence accession numbers used in the trnK intron and ITS phylogenetic analyses.

No.	Taxon	Collection locality	Geographic coordinates	trnK accession	ITS accession
1.1	*N. mirabilis* (Lour.) Druce	Nakhon Si Thammarat, Thailand	8.3475334°N, 99.7125974°E	C_AA462241.1	
1.2	*N. mirabilis* (Lour.) Druce	Satun, Thailand	7.0338056°N, 100.0282917°E	C_AA462242.1	
1.3	*N. mirabilis* (Lour.) Druce	Phang Nga, Thailand	8.8973437°N, 98.2698479°E	C_AA462240.1	
2.1	*N. mirabilis* var. *globosa* M.Catal.	Trang, Thailand	7.5153462°N, 99.5783642°E	C_AA462244.1	
2.2	*N. mirabilis* var. *globosa* M.Catal.	Phang Nga, Thailand	8.8965716°N, 98.2698505°E	C_AA462243.1	
3	*N. ampullaria* Jack	Songkhla, Thailand	7.0281542°N, 100.3743426°E	C_AA462229.1	
4	*N. gracilis* Korth.	Phatthalung, Thailand	7.2729117°N, 100.1920231°E	C_AA462233.1	
5	*N. rosea* M.Catal. & Kruetr.	Krabi, Thailand	8.0878785°N, 98.7756099°E	C_AA462245.1	
6	*N. kongkandana* M.Catal. & Kruetr.	Songkhla, Thailand	6.8845421°N, 100.9360950°E	C_AA462238.1	
7	*N. andamana* M.Catal.	Phang Nga, Thailand	8.9012219°N, 98.2701050°E	C_AA462230.1	
8	*N. suratensis* M.Catal.	Surat Thani, Thailand	9.1897309°N, 99.5407791°E	C_AA462248.1	
9	*N. kerrii* M.Catal. & Kruetr.	Satun, Thailand	6.5158120°N, 99.3110529°E	C_AA462237.1	
10	*N. krabiensis* Nuanlaong, Onsanit, Chusangr. & Suraninpong.	Krabi, Thailand	7.8992101°N, 99.3032899°E	C_AA462239.1	
11	*N. chang* M.Catal.	Trat, Thailand	12.0251375°N, 102.3411471°E	C_AA462232.1	
12.1	*N. kampotiana* Lecomte	Koh Kong, Cambodia	11.5146944°N, 103.1266111°E	C_AA462228.1	
12.2	*N. kampotiana* Lecomte	Trat, Thailand	11.9053270°N, 102.7988802°E	C_AA462236.1	
13	*N. thai* Cheek	Narathiwat, Thailand	5.8268963°N, 101.6901186°E	C_AA462249.1	

Table S7. *Nepenthes* taxa, collection localities, geographic coordinates, and sequence accession numbers used in the trnK intron and ITS phylogenetic analyses. (Count.)

No.	Taxon	Collection locality	Geographic coordinates	trnK accession	ITS accession
14	*N. sanguinea* Lindl.	Yala, Thailand	5.9153922°N, 101.3068112°E	C_AA462246.1	
15	*N. smilesii* Hemsl.	Loei, Thailand	16.8608208°N, 101.7505850°E	C_AA462247.1	
16	*N. thorelii* Lecomte	Tay Ninh, Vietnam	11.6386368°N, 105.9044199°E	C_AA462250.1	
17	*N. hirtella* Nuanlaong & Suran. *sp. nov*.	Krabi, Thailand	7.8407692°N, 99.2763659°E	C_AA462234.1	
18	*N. bokorensis* Mey	Kampot, Cambodia	10.6570162°N, 104.0009222°E	C_AA462231.1	
19	*N. holdenii* May	Pursat, Cambodia	12.0146950°N, 103.4084259°E	C_AA462235.1	
20	*N. rowaniae* F.M.Bailey	Queensland, Australia	11.0000000°S, 142.1833333°E	–	–

Table S8. Primers used for amplification of the trnK intron and ITS regions, including nucleotide sequences and melting temperatures (Tm).

Region	Primer	Sequences 5´–3´	T_m (°C)
trnk	2–*trnK*–3914F	GGGGTTGCTAACTCAACG G	58.8
trnk	Nep16–1270R	TTCGTCCAGTAGCGAATA G	54.5
trnk	Nep2–1060F	GGATTTGCATTCATTGTG G	52.4
trnk	16–*trnK*–2R	AACTAGTCGGATGGAGTA G	54.5
ITS	AITS1-F	AGAAGTCCACTGAACCTTATC	52.4
ITS	AITS4-R	CGCTTCTCCAGACTACAATTC	52.4

Primers for trnK intron amplification were adopted from Meimberg et al. (2006), whereas ITS primers were adopted from Alamsyah and Ito (2013).

Table S9. Sampling localities and geographic coordinates of 33 *Nepenthes mirabilis* accessions used for AFLP and ITS analyses.

No.	Accession code	Collection locality	Geographic coordinates
1	C1	Khuan Kalong District, Satun, Thailand	6.8655009°N, 100.0814832°E
2	C2	Ban Muang Thuan, Pak Phayun District, Phatthalung, Thailand	7.3106109°N, 100.2980606°E
3	C3	Khao Sun, Chawang District, Nakhon Si Thammarat, Thailand	8.4894887°N, 99.4721169°E
4	C4	Wang Mai Subdistrict, Pa Bon District, Phatthalung, Thailand	7.3472795°N, 100.1852391°E
5	C5	Su-ngai Kolok District, Narathiwat, Thailand	6.0692696°N, 101.9636822°E
6	C6	Thung Tam Sao Subdistrict, Songkhla, Thailand	6.9502265°N, 100.3841192°E
7	C7	Khok Pho District, Pattani, Thailand	6.6575250°N, 101.0943206°E
8	C8	Khao Dat Fa, Nakhon Si Thammarat, Thailand	9.1202652°N, 99.8266234°E
9	C9	Sakaom Subdistrict, Thepha District, Songkhla, Thailand	6.9136710°N, 100.9009859°E
10	C10	Ban Na Doem, Surat Thani, Thailand	8.8810575°N, 99.2607501°E
11	C11	Bang Saphan Noi District, Prachuap Khiri Khan, Thailand	11.0292861°N, 99.4604241°E
12	C12	Mueang District, Chumphon, Thailand	10.5263099°N, 99.1974727°E
13	C13	Sawi District, Chumphon, Thailand	10.2748955°N, 99.0819161°E
14	C14	Ban San Daeng, Khuan Kalong District, Satun, Thailand	7.0338056°N, 100.0282917°E
15	C15	Khlong Phon Subdistrict, Krabi, Thailand	7.8274837°N, 99.2765104°E
16	C16	Ban Thung Nui, Khuan Kalong District, Satun, Thailand	6.8589303°N, 100.1371994°E
17	C17	Don Pradu Subdistrict, Phatthalung, Thailand	7.2534418°N, 100.2760721°E
18	C18	Khlong Hoi Khong District, Songkhla, Thailand	6.8560389°N, 100.3741243°E
19	C19	Khuan Kalong District, Satun, Thailand	7.0361242°N, 100.0380519°E
20	C20	Bang Klam District, Songkhla, Thailand	7.0281524°N, 100.3743426°E
21	C21	Thung Khai Subdistrict, Trang, Thailand	7.4725398°N, 99.6383116°E
22	C22	Mueang District, Surat Thani, Thailand	9.0230710°N, 99.3575597°E
23	C23	Khun Thale Subdistrict, Surat Thani, Thailand	9.0757286°N, 99.3349816°E
24	C24	Khuan Lang Subdistrict, Songkhla, Thailand	6.9493719°N, 100.4172294°E
25	C25	Hala-Bala Forest, Yala, Thailand	5.8041449°N, 101.8175232°E
26	C26	Thung Khai Botanical Garden, Trang, Thailand	7.4723002°N, 99.6381154°E
27	C27	Rajamangala University of Technology Srivijaya, Trang, Thailand	7.5258668°N, 99.3145046°E
28	C28	Takua Pa District, Phang Nga, Thailand	8.7405429°N, 98.2802243°E
29	C29	Khlong Phanom National Park, Surat Thani, Thailand	8.7755717°N, 98.6919444°E
30	C30	Ko Kho Khao, Phang Nga, Thailand	8.8988760°N, 98.2705405°E
31	C31	Prince of Songkla University, Trang, Thailand	7.5153462°N, 99.5783642°E
32	C32	Ko Kho Khao, Phang Nga, Thailand	8.8999393°N, 98.2701798°E
33	C33	Mueang District, Nakhon Si Thammarat, Thailand	8.5424323°N, 99.9512157°E

Table S10. Pre-selective and selective AFLP primer sequences used for genetic analysis of Thai *Nepenthes mirabilis*.

Pre–selective primer

**primer**	**sequence (5'-3')**
ER-A	GACTGCGTACCAATTC**A**
MS-C	GACTGCGTACCAATTC**C**

Selective primer

**primer**	**sequence (5'-3')**
ER-AT	5'GACTGCGTACCAATTC**AT** 3'
ER-AA	5'GACTGCGTACCAATTC**AA** 3'
ER-AC	5'GACTGCGTACCAATTC**AC** 3'
ER-AG	5'GACTGCGTACCAATTC**AG** 3'
ER- AGA	5'GACTGCGTACCAATTC**AGA** 3'
ER- ACC	5'GACTGCGTACCAATTC**ACC** 3'
ER- AAG	5'GACTGCGTACCAATTC**AAG** 3'
ER- AAC	5'GACTGCGTACCAATTC**AAC** 3'
ER- ACA	5'GACTGCGTACCAATTC**ACA** 3'
ER- AGC	5'GACTGCGTACCAATTC**AGC** 3'
ER- ATG	5'GACTGCGTACCAATTC**ATG** 3'
ER- AGG	5'GACTGCGTACCAATTC**AGG** 3'
MS-CAA	5'GATGAGTCCTGAGTAA**CAA** 3'
MS-CAT	5'GATGAGTCCTGAGTAA**CAT** 3'
MS-CAC	5'GATGAGTCCTGAGTAA**CAC** 3'
